# Assessing socioeconomic status through dental and associated tissue characteristics: A cross-sectional study for human identification

**DOI:** 10.12688/f1000research.161533.1

**Published:** 2025-02-18

**Authors:** CEENA DENNY, Srikant Natarajan, Jasmine Jophy, Nandita KP, Amita Juanita Lewis, Shwetha Yellapurkar

**Affiliations:** 1 Department of Oral Medicine Radiology, Manipal College of Dental Sciences, Mangalore, Manipal Academy of Higher Education, Manipal, Karnataka, 576104, India; 2 Department of Oral Pathology and Microbiology, Manipal College of Dental Sciences, Mangalore, Manipal Academy of Higher Education, Manipal, Karnataka, 576104, India

**Keywords:** Socioeconomic Status (SES), Dental Health Indicators, Human Identification

## Abstract

**Background:**

Dental identification methods are employed to determine a person’s identity in forensic odontology. Additionally, analyzing a person’s teeth can provide insights into their socioeconomic status, which may assist in the process of identification. Our study aimed to assess the correlation between dental health status and SES among individuals, with a focus on identifying predictors of SES based on dental health indicators

**Methods:**

A cross-sectional study was conducted to evaluate the correlation between dental health status and socioeconomic status among 135 individuals. who had visited the Department of Oral Medicine for various forms of dental care. SES was measured according to a modified Kuppuswamy scale. The associations between SES and dental health indicators were analyzed using chi-square tests for categorical variables and t-tests for continuous variables.

**Results:**

Dental caries is more common in lower SES groups, attributed to factors like poor nutrition and hygiene. Partial and complete edentulism are also more prevalent in lower SES populations, often due to financial constraints and lack of awareness, leading to extractions rather than restorations. However, urban populations, regardless of SES, show greater utilization of dental restorations, likely due to the concentration of dentists in cities and access to community-based dental camps offering low-cost treatments. Periodontal diseases further reflect SES disparities. Studies show that individuals from lower SES backgrounds report more severe periodontal issues, such as gingival swelling, while higher SES groups report milder symptoms like gingival bleeding, indicating earlier intervention.

**Conclusion:**

SES plays a vital role in shaping health and lifestyle outcomes. The findings in our study demonstrate the value of incorporating SES indicators, such as education, occupation, marital status, and residence, into forensic investigations to enhance the precision of identification processes.

## Introduction

The impact of socioeconomic status (SES) on oral health is indeed significant. People with low SES tend to have higher rates of dental issues, often due to limited access to essential resources such as healthcare and proper nutrition. In India, research has shown that SES influences oral health indirectly, with poor SES negatively affecting oral health outcomes. ^1^ Socioeconomic status plays a vital role in mediating the relationship between dental maturation and access to crucial resources necessary for healthy growth and development. Comparing patients from different socioeconomic backgrounds can provide insights into disparities in access to resources such as nutrition and healthcare. ^2^ Assessing a patient’s SES through their teeth, dental treatments, and oral hygiene, as well as demographic information, occupation, and education level can offer valuable information. The idea of the project is to reverse engineer the prediction of SES on the basis of dental and associated findings. The data used for human identification is surrogately classified based on SES such as school admissions, ration cards, car rentals, travel itineraries etc. The estimation of SESs can prove to be an efficient tool to narrow down the relevant data set from which human identification is possible. Hence, this study aims to explore the connection between SES and tooth structure, shedding light on how these factors are intertwined in influencing oral health. ^3^ This study aims to assess the correlation between dental health status and SES among individuals, with a focus on identifying predictors of SES based on dental health indicators

## Methods

A cross-sectional study was conducted to evaluate the correlation between dental health status and socioeconomic status among individuals. The sample of the study was composed of a total of 135 subjects who had visited the Department of Oral Medicine and Radiology for various forms of dental care. The participants were initially briefed about the study. Once the subject was willing to participate in the study, the principal investigator entered the details in the information sheet. The study was conducted after the approval of the Institutional Ethics Committee (Protocol no. 24105,13/8/2024). The study included adults aged 18-65 years. Participants were recruited using convenience sampling from various urban and rural locations.

### Sample size

Based on the article by Ramamoorthy J and Mahalakshmi J (2022) ^4^ the proportion of patients with caries status in the low-income group was reported to be 21% With an alpha of 5% the corresponding z value is Z=-1. Using the formula n= ((n=((Z _(α/2)_/d) ^2^)(p(1-p)))) with the minimum percentage difference to be deemed clinically significant at 7%, the sample size]required would be 131 for the study to ensure a diverse sample.

The inclusion criteria for the study were that all the subjects were of Indian nationality and without any developmental disorders that could affect normal tooth development. The exclusion criteria were those subjects with trismus, being completely edentulous or suffering from systemic diseases that can affect dental mineralization. The required data such as demographic, socioeconomic, and health- related information were obtained through a structured questionnaire. All the entries were recorded by a single observer. SES was measured according to a modified Kuppuswamy scale (2024). ^5^ A detailed oral examination was conducted, along with an evaluation of tooth wear according to Roehl JC et al. (2021) ^6^ and the findings were entered into an Excel sheet. ^6^

To address potential sources of bias in the study, several measures were implemented. Participants were recruited using convenience sampling from diverse urban and rural locations to ensure representation across different socioeconomic backgrounds. A single observer recorded all data to minimize inter-observer variability, and a structured questionnaire was used to standardize the collection of demographic, socioeconomic, and health-related information. The oral examinations were conducted following standardized protocols to maintain consistency, and the SES was assessed using the validated Modified Kuppuswamy Scale (2024). ^5^ Additionally, exclusion criteria were clearly defined to eliminate confounding factors such as systemic diseases, developmental disorders, or edentulism that could skew dental health outcomes. These measures aimed to reduce selection, measurement, and information bias in the study.

In the study, missing data were addressed through careful measures to minimize its occurrence and manage it appropriately if it arose. During data collection, participants were briefed about the importance of providing complete and accurate information, and every effort was made to ensure comprehensive responses to the structured questionnaire. If any incomplete responses or missing entries were identified, participants were contacted (where possible) to clarify or complete the missing information.

### Statistical analysis

The SPSS 20.0 (IBM Chicago) (Statistical Package for the Social Sciences) package was used to analyze the data. Descriptive statistics were used to summarize the demographic and dental health characteristics of the participants. The associations between SES and dental health indicators were analyzed using chi-square tests for categorical variables and t-tests for continuous variables. Binary logistic regression analysis was used to predict higher SES in relation to findings of the tooth morphology and diseases. A p-value of <0.05 was considered to indicate statistical significance described with a 95% confidence interval.

## Results

A total of 135 participants aged 18 and above were included in the study. Of these, 73 (54.1%) were female and 62 (45.9%) were male. Additionally, 96 (71.1%) were married and 39 (28.9%) were single. 123 (91.1%) participants were from Karnataka, while 12 (8.9%) were from outside the state. 92 (68.1%) participants lived in urban areas, and 43 (31.9%) resided in rural areas. Most of the participants were from the higher SES (66/%) when compared to lower SES (34%).

For **gender**, a greater proportion of females were in the higher SES group (65.2%) than in the lower SES group (48.3%), although this difference was not statistically significant (p = 0.062). **Marital status** revealed significant differences (p = 0.022), with a greater percentage of married individuals in the lower SES group (77.5%) than in the higher SES group (58.7%). **Smokeless tobacco use** was more common in the lower SES group (12.4%) than in the higher SES group (2.2%), with this difference being statistically significant (p = 0.049). **Education** also showed significant variability across SES groups (p = 0.004), with the higher SES group showing a greater proportion of professional degrees (23.9%) compared to the lower SES group (4.5%). **Occupation** displayed the most significant association (p < 0.001), with a greater concentration of professionals in the higher SES group (28.3%) than in the lower SES group, where semiskilled and unskilled occupations were more common.

Most other variables, including diet, smoking habits, urban-rural residence, and various oral health conditions, were not significantly different between SES groups, with p-values generally above 0.05, suggesting that there was no strong association between these factors and SES in this sample.

The observations related to oral manifestations and their association with socioeconomic status (SES) reveal unique patterns. The prevalence of missing teeth is higher in lower SES groups (43.8%) compared to higher SES groups (28.3%), though the difference is not statistically significant (p = 0.078). The occurrence of dental restorations is also more frequent in the lower SES group (44.9%) than in the higher SES group (34.8%), but the difference remains insignificant (p = 0.256). Dental caries is slightly more common in the lower SES group (91%) than in the higher SES group (87%), with no significant difference (p = 0.464). Gingivitis shows a higher prevalence in the higher SES group (43.5%) compared to the lower SES group (34.8%), though this difference is not statistically significant (p = 0.326). Similarly, periodontitis is slightly more common in the higher SES group (23.9%) than in the lower SES group (22.5%), with no significant difference observed (p = 0.85).

Tooth wear patterns also vary between SES groups. Buccal tooth wear is higher in the lower SES group (36%) compared to the higher SES group (28.3%), but this difference is not significant (p = 0.369). Palatal tooth wear and occlusal tooth wear also do not show significant differences between SES groups, with p-values of 0.686 and 0.488, respectively. Regarding tooth surface appearance, the absence of abnormalities is slightly more common in the higher SES group (43.5%) than in the lower SES group (29.2%), though not significant (p = 0.348). Cracks in enamel are rare across both groups, and the rate of enamel and dentin wear remains similar (p = 0.688).

In terms of restoration outcomes, nil restoration cases are slightly more frequent in the higher SES group (58.7%) compared to the lower SES group (51.7%), but this difference is not significant (p = 0.735). Patterns of attrition, such as rounding of cusps and grooves or shiny facets, do not significantly differ between SES groups (p = 0.248). Attrition patterns like occlusal cupping or incisal grooving are rare and do not show a notable SES-related trend.

Soft tissue changes such as impressions on the cheek are more prevalent in the higher SES group (19.6%) than in the lower SES group (7.9%), but this difference is not statistically significant (p = 0.214). Pathological signs, including pain and sensitivity (50% in higher SES vs. 47.7% in lower SES) and functional problems, show minimal differences across SES groups, with no significant p-values. Similarly, deterioration of dental aesthetics does not vary significantly between groups (p = 0.896). These observations highlight that while some oral health parameters show variations between SES groups, most differences are not statistically significant ( Table 1).

**Table 1.** Chi square test to compare the SES category I/II vs Category III/IV for all variables.

	Categories	N	SES_Upper_Lower		Chi square	P value
			SES score III/IV (N (%))	SES score I/II (N (%))		
Sex	Female	73	43 (48.3)	30 (65.2)	3.489	0.062
	Male	62	46 (51.7)	16 (34.8)		
Marital Status	Married	96	69 (77.5)	27 (58.7)	5.236	**0.022**
	Single	39	20 (22.5)	19 (41.3)		
Location	Karnataka	123	83 (93.3)	40 (87)	1.487	0.223
	Non karnataka	12	6 (6.7)	6 (13)		
Urban/Rural	Urban	92	59 (66.3)	33 (71.7)	0.415	0.52
	Rural	43	30 (33.7)	13 (28.3)		
Habits-Alcohol	Absent	127	84 (94.4)	43 (93.5)	0.044	0.833
	Present	8	5 (5.6)	3 (6.5)		
Smoking	Absent	123	81 (91)	42 (91.3)	0.003	0.955
	Present	12	8 (9)	4 (8.7)		
Smokeless	Absent	123	78 (87.6)	45 (97.8)	3.885	**0.049**
	Present	12	11 (12.4)	1 (2.2)		
Diet	Non-Veg	7	5 (5.6)	2 (4.3)	0.1	0.752
	Veg	128	84 (94.4)	44 (95.7)		
Education	Illiterate	0	0 (0)	0 (0)	17.427	**0.004**
	Primary School Certificate	6	6 (6.7)	0 (0)		
	Middle School Certificate	10	9 (10.1)	1 (2.2)		
	High School Certificate	29	22 (24.7)	7 (15.2)		
	Intermediate or Post High School Diploma	32	20 (22.5)	12 (26.1)		
	Graduate or Postgraduate	43	28 (31.5)	15 (32.6)		
	Professional Degree	15	4 (4.5)	11 (23.9)		
Occupation	Unemployed	14	11 (12.4)	3 (6.5)	46.471	**<0.001**
	Unskilled Worker	8	8 (9)	0 (0)		
	Semi Skilled Worker	17	17 (19.1)	0 (0)		
	Skilled Worker	16	14 (15.7)	2 (4.3)		
	Clerical/Shop owner/Farm	32	23 (25.8)	9 (19.6)		
	Semi Professional	34	15 (16.9)	19 (41.3)		
	Professional (White Collar)	14	1 (1.1)	13 (28.3)		
Oral manifestations
Missing teeth	Absent	83	50 (56.2)	33 (71.7)	3.1	0.078
	Present	52	39 (43.8)	13 (28.3)		
Restoration	Absent	79	49 (55.1)	30 (65.2)	1.29	0.256
	Present	56	40 (44.9)	16 (34.8)		
Dental caries	Absent	14	8 (9)	6 (13)	0.536	0.464
	Present	121	81 (91)	40 (87)		
Gingivitis	Absent	84	58 (65.2)	26 (56.5)	0.965	0.326
	Present	51	31 (34.8)	20 (43.5)		
Periodontitis	Absent	104	69 (77.5)	35 (76.1)	0.036	0.85
	Present	31	20 (22.5)	11 (23.9)		
Tooth Wear (Buccal)	Absent	90	57 (64)	33 (71.7)	0.808	0.369
	Present	45	32 (36)	13 (28.3)		
Tooth Wear (Palatal)	Absent	112	73 (82)	39 (84.8)	0.163	0.686
	Present	23	16 (18)	7 (15.2)		
Tooth Wear (Occlusal)	Absent	76	52 (58.4)	24 (52.2)	0.482	0.488
	Present	59	37 (41.6)	22 (47.8)		
Tooth Surface	Absent	46	26 (29.2)	20 (43.5)	3.301	0.348
	Smooth Silky Shiny appearance	51	36 (40.4)	15 (32.6)		
	Increased incisal translucency	21	16 (18)	5 (10.9)		
	No plaque, Discoloration or tartar	17	11 (12.4)	6 (13)		
Tooth Defect	Absent	61	38 (42.7)	23 (50)	1.475	0.688
	Enamel and dentin wear at same rate	48	32 (36)	16 (34.8)		
	Fracture of cusps or restorations	24	18 (20.2)	6 (13)		
	Cracks with enamel	2	1 (1.1)	1 (2.2)		
Distribution of tooth wear	Nil	56	35 (39.3)	21 (45.7)	1.364	0.714
	Wear on occluding surfaces	48	31 (34.8)	17 (37)		
	Wear on non occluding surfaces	26	19 (21.3)	7 (15.2)		
	located at cervical areas of teeth	5	4 (4.5)	1 (2.2)		
Restoration	Nil	73	46 (51.7)	27 (58.7)	0.616	0.735
	Clean non tarnished appearance	45	31 (34.8)	14 (30.4)		
	Raised restoration	17	12 (13.5)	5 (10.9)		
Attrition pattern	Nil	74	45 (50.6)	29 (63)	5.404	0.248
	Rounding of cusps and grooves	40	28 (31.5)	12 (26.1)		
	shiny facets flat and glossy	13	8 (9)	5 (10.9)		
	occlusal cupping	5	5 (5.6)	0 (0)		
	Incisal grooving	3	3 (3.4)	0 (0)		
Soft Tissue Changes	Nil	114	78 (87.6)	36 (78.3)	4.482	0.214
	Impressions on cheek	16	7 (7.9)	9 (19.6)		
	Impression on tongue	4	3 (3.4)	1 (2.2)		
Pathological Sign	Nil	40	26 (29.5)	14 (30.4)	1.086	0.896
	Pain and sensitivity	65	42 (47.7)	23 (50)		
	Functional problems	13	9 (10.2)	4 (8.7)		
	Crumbling of dental hard tissue and restoration	6	5 (5.7)	1 (2.2)		
	Deterioration of aesthetic appearance	10	6 (6.8)	4 (8.7)		

Table 1 presents a comparative analysis of various demographic, behavioral, and clinical characteristics across different SES groups, SES III/IV (lower SES) and SES I/II (higher SES).

Binary logistic regression analysis was performed to predict higher SES in relation to findings of tooth morphology and diseases. In this analysis, the outcome being studied is the likelihood of belonging to a higher socioeconomic status (SES) score of III or IV than to a lower SES score of I or II. The regression results highlight several key factors influencing SES classification ( Table 2).

**Table 2.** Binarly logistic regression analysis to predict the higher SES in relation to findings of the tooth morphology and diseases.

Variable	Category (Reference)	B	S.E.	Wald	df	p-value	Odds Ratio	95% C.I. for Odds Ratio (Lower)	95% C.I. for Odds Ratio (Upper)
**SEX**	Male (Female)	-1.122	0.581	3.731	1	0.053	0.326	0.104	1.017
**MARITAL STATUS**	Single (Married)	1.871	0.733	6.517	1	***0.011***	6.498	1.544	27.337
**LOCATION**	Single (Karnataka)	0.789	0.887	0.793	1	0.373	2.202	0.387	12.516
**URBAN/RURAL**	Rural (Urban)	0.009	0.599	0.000	1	0.988	1.009	0.312	3.266
**HABIT**	Present (Absent)	0.285	1.219	0.055	1	0.815	1.330	0.122	14.506
**SMOKING**	Present (Absent)	1.538	1.023	2.260	1	0.133	4.653	0.627	34.546
**SMOKELESS**	Present (Absent)	-1.396	1.295	1.161	1	0.281	0.248	0.020	3.136
**DIET**	Veg (Non Veg)	1.798	1.283	1.964	1	0.161	6.039	0.488	74.695
**CARIES RELATED PROBLEM**	Present (Absent)	0.139	0.873	0.026	1	0.873	1.150	0.208	6.360
**PERIODONTIUM RELATED PROBLEM**	Present (Absent)	0.541	0.549	0.969	1	0.325	1.717	0.585	5.041
**ANY ASSOCIATED PROBLEM**	Present (Absent)	-19.698	15512.448	0.000	1	0.999	0.000	0.000	—
**TOOTH WEAR BUCCAL**	Present (Absent)	0.031	0.643	0.002	1	0.962	1.031	0.292	3.638
**TOOTH WEAR PALATAL**	Present (Absent)	0.212	0.900	0.055	1	0.814	1.236	0.212	7.209
**TOOTH WEAR OCCLUSAL**	Present (Absent)	0.786	0.617	1.622	1	0.203	2.194	0.655	7.353
**TOOTH SURFACE**		—	—	7.693	3	0.053	—	—	—
**TOOTH SURFACE(1)**	Smooth Silky Shiny	-2.507	1.074	5.449	1	***0.020***	0.081	0.010	0.669
**TOOTH SURFACE(2)**	Incisal Translucency	-3.021	1.186	6.486	1	***0.011***	0.049	0.005	0.499
**TOOTH SURFACE(3)**	No Plaque/Tartar	-1.568	0.927	2.859	1	0.091	0.208	0.034	1.284
**TOOTH DEFECT**		—	—	1.641	3	0.650	—	—	—
**TOOTH DEFECT(1)**	Enamel & Dentin Wear	-0.839	0.807	1.083	1	0.298	0.432	0.089	2.100
**TOOTH DEFECT(2)**	Fracture of Cusps	-0.953	0.838	1.291	1	0.256	0.386	0.075	1.995
**TOOTH DEFECT(3)**	Cracks with Enamel	18.731	20298.220	0.000	1	0.999	136360212.606	0.000	—
**DISTRIBUTION**		—	—	3.016	3	0.389	—	—	—
**DISTRIBUTION(1)**	Wear on Occluding Surfaces	0.982	0.939	1.095	1	0.295	2.671	0.424	16.815
**DISTRIBUTION(2)**	Wear on Non-Occluding Surfaces	0.198	1.191	0.028	1	0.868	1.219	0.118	12.582
**DISTRIBUTION(3)**	Cervical Area	-1.199	1.667	0.517	1	0.472	0.302	0.012	7.908
**RESTORATION**		—	—	1.190	2	0.551	—	—	—
**RESTORATION(1)**	Clean Non-Tarnished	0.090	0.684	0.017	1	0.895	1.094	0.286	4.180
**RESTORATION(2)**	Raised Restoration	0.992	0.963	1.062	1	0.303	2.697	0.409	17.792
**SOFT AND HARD TISSUE**		—	—	4.045	3	0.257	—	—	—
**SOFT AND HARD TISSUE(1)**	Impressions on Cheek	1.656	0.823	4.045	1	***0.044***	5.237	1.043	26.292
**SOFT AND HARD TISSUE(2)**	Impression on Tongue	0.587	1.669	0.124	1	0.725	1.799	0.068	47.446
**AGE**	—	0.022	0.019	1.334	1	0.248	1.022	0.985	1.062
**Constant**	—	-3.681	2.045	3.242	1				

**Sex** shows a marginal association, where males have lower odds of being in the higher SES category (OR = 0.326, p = 0.053). **Marital status** is significantly associated with SES, as single individuals are 6.5 times more likely to be in the higher SES group than are married individuals (OR = 6.498, p = 0.011). **Location/**residency **(Urban/Rural)** did not significantly show any effect on SES (OR = 2.202, p = 0.373 and OR = 1.009, p = 0.988, respectively). The presence of certain **habits** also did not significantly affect SES (OR = 1.330, p = 0.815), **smoking** (OR = 4.653, p = 0.133) or **diet** (vegetarians vs. nonvegetarians, OR = 6.039, p = 0.161). Similarly, the **presence of caries-related problems** was not strongly associated with SES (OR = 1.150, p = 0.873).

Among the dental variables, specific **tooth surface** conditions were significantly associated: individuals with “Smooth Silky Shiny” tooth surfaces were less likely to belong to a higher SES group (OR = 0.081, p = 0.020), as were those with **increased incisal translucency** (OR = 0.049, p = 0.011). **Soft and hard tissue** impressions on the cheek significantly increased the odds of being in the higher SES category (OR = 5.237, p = 0.044). Finally, age and other specific oral conditions such as tooth defects **Tooth defects** and **tooth wear** were not significantly associated with SES status.

In summary, single marital status and cheek impressions are associated with higher odds of belonging to a higher SES group, whereas certain tooth surface conditions, particularly smooth and translucent surfaces, are linked with lower odds of higher SES classification.

## Discussion

Socioeconomic status (SES) represents the relative position of individuals, families, or groups within a societal hierarchy based on access to valued goods like wealth, social recognition, and privileges. ^7^ It is a multidimensional construct encompassing income, educational attainment, occupation, and quality of personal care, including oral health maintenance. ^8^ SES significantly impacts health outcomes, with lower SES associated with greater health issues, including dental problems due to limited healthcare access, poor nutrition, and low health literacy. ^4, 9^ Teeth and their associated characteristics, along with demographic information, can provide insights into an individual’s SES, aiding in identification by narrowing potential datasets on the basis of socioeconomic factors which could aid in forensic investigations. ^10^

The modified Kuppuswamy scale, introduced in 1981 and updated in 2024, categorizes SES based on education, occupation, and monthly income of the head of the family. It classifies individuals into upper, upper-middle, lower-middle, upper-lower, or lower classes, but limitations exist, particularly in distinguishing joint versus nuclear families and its urban-centric focus. ^5, 11^ SES also influences education, with higher SES groups more likely to attain professional degrees due to fewer financial constraints and greater awareness of the benefits of education. Occupation is another critical factor, with higher SES groups predominantly engaged in professional roles, while lower SES groups often work in semi-skilled or unskilled jobs.

Marital status: In our study, we found that individuals with lower socioeconomic status (SES) were more prevalent in terms of marital status. Individuals with higher SES, delay marriage and childbirth to focus on their education and careers. ^12^ Marital status can be an indirect indicator of socioeconomic position, as it can be associated with factors such as income, social support, and access to healthcare.

Residence (Urban/Rural): It is generally understood that a place of residence (urban/rural) can indirectly influence SES. The reasons are as follows: a) access to education and employment opportunities often differs between urban and rural areas, b) cost of living and income levels can vary significantly between these locations and c) availability and quality of healthcare services may also differ. These factors can contribute to socioeconomic disparities between urban and rural populations. Therefore, while the sources focus primarily on the impact of individual-level socioeconomic factors, it is important to acknowledge that a place of residence can play a complex and multifaceted role in shaping both SES and health outcomes. ^13^ Most of the study participants were from urban areas, as our institution is situated in an urban environment, regardless of whether they belong to higher or lower socioeconomic status even though it was statistically insignificant.

**Education:** Research shows that socioeconomic status (SES) plays a significant role in educational outcomes, with the higher SES group showing a greater proportion of professional degrees than the lower SES group. Notably, parents with higher SES are more likely to send their children to attain higher degrees. This could be because they can afford as they do not have any financial restraints and are more knowledgeable about education than people of lower SES. ^14^

**Occupation:** Displayed the most significant association, with a greater concentration of professionals in the higher SES group than in the lower SES group, where semiskilled and unskilled occupations were more common.

**Smokeless tobacco:** Various studies have highlighted the correlation between SES and health behaviors, such as smokeless tobacco use, which is more prevalent among lower SES groups. ^15, 16^ Tobacco use is closely related to SES. ^17^ The consumption of tobacco is more prevalent among the lower impoverished in India. ^18^ The various oral health issues observed associated with the use of tobacco products were staining of teeth, tooth wear, tooth loss, dental caries, edentulousness. Additionally, tobacco use contributes to the buildup of plaque and calculus, with exposure of tooth root surface, periodontal pocket formation, increasing the risk of periodontitis, especially in the front teeth. Other issues include tobacco pouch keratosis, various potentially malignant oral disorders, and an increased risk of oral squamous cell carcinoma. ^19, 20^It is also noted that tobacco consumption is declining more rapidly among people with higher SES than among those with when compared to the lower SES ^21^ reflecting better awareness and access to cessation resources.

**Oral findings:** Dental caries (DC): More commonly seen in people with lower SES. ^4, 22, 23^ This could be because Individuals with lower SES often have poor oral hygiene practices and increase susceptibility to dental caries (DC). Though statistically insignificant in some studies, lower SES groups consistently exhibit higher dental caries prevalence, which is linked to factors like poor education, income constraints, and lack of awareness about oral health.

**Edentulism:** Partial or complete edentulism was more common in individuals with lower SES, these findings are similar to those of our study but statistically insignificant. They attributed the reason for edentulousness to lack of awareness and financial constraints, education and occupation as the root cause, hence, they would have opted for extraction of teeth. ^24, 25^

**Restorations:** Although statistically insignificant in our study, we observed that even individuals with lower SES had gotten tooth restored. This could be due to most of our patients were from urban area where almost the 3/4 ^th^ dentist of India reside. ^26^ Dental institutions in India provide community-based dental camps in rural areas which spread awareness about dental health hand also provide dental treatment either free of cost or at minimal rates.

**Periodontal diseases:** Individuals with lower socioeconomic status tended to report more advanced symptoms of periodontal disease, including gingival swelling, whereas those with higher socioeconomic status were more likely to report early-stage symptoms, such as gingival bleeding. ^27– 29^ These findings were similar to those of our study.

**Tooth wear:** The lower socioeconomic group was found to be the most affected by tooth wear. ^30, 31^ These findings were in line with our study although they were statistically insignificant. Possible explanations for this disparity include a lack of awareness, knowledge, and education regarding the factors that contribute to tooth wear. ^32^

Smooth silky shiny teeth are a sign of mineral loss and incisal translucency is a sign of enamel loss. Individuals with low socioeconomic status are more susceptible to demineralization because of differing priority needs, lower levels of education, and limited access to preventive programs. ^33^ These findings are similar to our observations. A systematic review and meta-analysis of observational studies performed by Entezami S(2021) revealed that tooth wear was greater in individuals with higher SES due to increased consumption of juices with high acidic contents. ^34^

**Soft tissue changes:** Impressions on the cheek significantly increase the odds of being in the higher SES category. This could be due to the stress related to higher SES as society rapidly evolves, as individuals encounter a variety of stressors, including those related to work, family, relationships, finances, socio-cultural issues, and health. ^35^ Hashibe M, Jacob BJ et al (2003) ^36^ reported that people with high income could have more people in their household especially in the Indian population which could affect cleanliness which lead to development of fungal and viral lesions, however,in their study they reported that oral lesions were more common in people with lower SES which was in accordance with the study done by Taruna T, Singh YP, et al. (2023). ^37^

Dental health indicators provide significant insights into SES. For example, dental caries is more common in lower SES groups, attributed to factors like poor nutrition and hygiene. Partial and complete edentulism are also more prevalent in lower SES populations, often due to financial constraints and lack of awareness, leading to extractions rather than restorations. However, urban populations, regardless of SES, show greater utilization of dental restorations, likely due to the concentration of dentists in cities and access to community-based dental camps offering low-cost treatments. Periodontal diseases further reflect SES disparities. Studies show that individuals from lower SES backgrounds report more severe periodontal issues, such as gingival swelling, while higher SES groups report milder symptoms like gingival bleeding, indicating earlier intervention.

## Conclusion

Overall, SES plays a vital role in shaping health and lifestyle outcomes, influencing access to education, healthcare, and occupational opportunities. By understanding the socioeconomic roots of health disparities, researchers and policymakers can better target interventions to improve health equity. These findings also demonstrate the value of incorporating SES indicators, such as education, occupation, marital status, and residence, into forensic investigations to enhance the precision of identification processes.

### Ethical considerations

Ethics approval and consent to participate: Approval for the study was taken prior to conducting the study from Institutional Ethics Committee, Manipal College of Dental Sciences, Mangalore (Protocol no. 24105,13/8/2024). Written informed consent for publication of their details was obtained from the study participants.

## Data Availability

Figshare repository: Assessing Socioeconomic Status Through Dental and Associated Tissue Characteristics: A Cross-Sectional Study for Human Identification, https://www.doi.org/10.6084/m9.figshare.28308611.v2. ^38^ The project contains the following underlying data:
1. SES EXCEL 1.xlsx. Data are available under the terms of the Creative Commons Attribution 4.0 International license (CC-BY 4.0).
