# Supplementary material for: Assessing socioeconomic status through dental and associated tissue characteristics: A cross-sectional study for human identification
Source: F1000Res. 2025 Mar 18;14:220. Originally published 2025 Feb 18. [Version 3] doi: 10.12688/f1000research.161533.3 (PMC11907184; doi:10.12688/f1000research.161533.3)
Supplement: Peer review history

# Reviewer report: Reviewer response for version 2

**DOI:** 10.5256/f1000research.178770.r369920

**Published:** 2025-03-13

**Authors:** Korikkar Mahaling Veena

**Affiliations:** 1 Oral medicine and Radiology, Yenepoya Dental College, Yenepoya (deemed to be) University, Mangaluru, Karnataka, India

The authors have considered the suggestion given and incorporated them in the manuscript and I have no further comments to make.

Is the work clearly and accurately presented and does it cite the current literature?

Yes

If applicable, is the statistical analysis and its interpretation appropriate?

I cannot comment. A qualified statistician is required.

Are all the source data underlying the results available to ensure full reproducibility?

No source data required

Is the study design appropriate and is the work technically sound?

Yes

Are the conclusions drawn adequately supported by the results?

Partly

Are sufficient details of methods and analysis provided to allow replication by others?

Yes

Reviewer Expertise:

Potentially malignant disorders of the oral mucosa

I confirm that I have read this submission and believe that I have an appropriate level of expertise to confirm that it is of an acceptable scientific standard.

# Reviewer report: Reviewer response for version 2

**DOI:** 10.5256/f1000research.178770.r369921

**Published:** 2025-03-12

**Authors:** Selvamani M

**Affiliations:** 1 Mahe Institute of Dental Sciences and Hospital, Mahé, India

The article is excellently written, and I have no additional comments or suggestions to make.

Is the work clearly and accurately presented and does it cite the current literature?

Yes

If applicable, is the statistical analysis and its interpretation appropriate?

Yes

Are all the source data underlying the results available to ensure full reproducibility?

Yes

Is the study design appropriate and is the work technically sound?

Yes

Are the conclusions drawn adequately supported by the results?

Yes

Are sufficient details of methods and analysis provided to allow replication by others?

Yes

Reviewer Expertise:

Forensic Odontology, Forensic Science, Odontogenic Cyst & Tumors

I confirm that I have read this submission and believe that I have an appropriate level of expertise to confirm that it is of an acceptable scientific standard.

# Reviewer report: Reviewer response for version 1

**DOI:** 10.5256/f1000research.177572.r367471

**Published:** 2025-02-28

**Authors:** Selvamani M

**Affiliations:** 1 Mahe Institute of Dental Sciences and Hospital, Mahé, India

The article effectively aligns with the study's objective, ensuring that the conclusions drawn are well-supported by the results obtained. The research presents an innovative approach with significant implications, particularly in the field of human identification through the assessment of socio-economic status. This study contributes valuable insights and has the potential to enhance forensic and social research methodologies.

 My decision is approved with reservations.

 Here are a few suggestions I would like to forward for the author:

 a. **Participation Selection Criteria:**

 Although the author has outlined the inclusion and exclusion criteria, it would be helpful to provide a rationale for these choices. For instance, what was the reasoning behind limiting the age range to 18-65 years?

 b. **Statistical Analysis:**

 It would be beneficial to justify the selection of the statistical methods used. For example, an explanation of why the chi-square test, t-test, and binary logistic regression were deemed appropriate for the data would add clarity.

 c. **Tables and Figures:**

 Consider incorporating visual aids, such as graphs or charts, to present key data for easier comprehension. For instance, a bar chart showing the differences in dental health parameters across SES groups could improve understanding.

Is the work clearly and accurately presented and does it cite the current literature?

Yes

If applicable, is the statistical analysis and its interpretation appropriate?

Yes

Are all the source data underlying the results available to ensure full reproducibility?

Yes

Is the study design appropriate and is the work technically sound?

Yes

Are the conclusions drawn adequately supported by the results?

Yes

Are sufficient details of methods and analysis provided to allow replication by others?

Yes

Reviewer Expertise:

Forensic Odontology, Forensic Science, Odontogenic Cyst & Tumors

I confirm that I have read this submission and believe that I have an appropriate level of expertise to confirm that it is of an acceptable scientific standard, however I have significant reservations, as outlined above.

# Response

**Published:** 2025-02-27

**Authors:** CEENA DENNY

**Affiliations:** Manipal College of Dental Sciences,Mangalore,Manipal Academy of Higher Education,Manipal-575001, India

I have  added a sentence and highlighted it in  under the methods section

 The family income was chosen to assess socioeconomic status instead of that of the head of the household.

# Reviewer report: Reviewer response for version 1

**DOI:** 10.5256/f1000research.177572.r367473

**Published:** 2025-02-22

**Authors:** Korikkar Mahaling Veena

**Affiliations:** 1 Oral medicine and Radiology, Yenepoya Dental College, Yenepoya (deemed to be) University, Mangaluru, Karnataka, India

Socioeconomic status is a well documented determinant of oral health, as it influences access to dental care, oral hygiene practices and diet. In case of females, their oral health status will be influenced by SEC of parent before marriage which is very important factor in relation to dental caries and periodontal health. The study's demographic distribution, with 54.1% females and 71.1% married participants, their oral health must have had influence by both parents SEC (before marriage) as well as spouse (currently). So it's a suggestion if it can be mentioned in inclusion criteria if both income was taken into consideration or not and then can be discussed about its impact in the discussion part.

Is the work clearly and accurately presented and does it cite the current literature?

Yes

If applicable, is the statistical analysis and its interpretation appropriate?

I cannot comment. A qualified statistician is required.

Are all the source data underlying the results available to ensure full reproducibility?

No source data required

Is the study design appropriate and is the work technically sound?

Yes

Are the conclusions drawn adequately supported by the results?

Partly

Are sufficient details of methods and analysis provided to allow replication by others?

Yes

Reviewer Expertise:

Potentially malignant disorders of the oral mucosa

I confirm that I have read this submission and believe that I have an appropriate level of expertise to confirm that it is of an acceptable scientific standard, however I have significant reservations, as outlined above.
